# Mechanisms of Systolic Cardiac Dysfunction in PP2A, PP5 and PP2AxPP5 Double Transgenic Mice

**DOI:** 10.3390/ijms22179448

**Published:** 2021-08-31

**Authors:** Mara-Francine Dörner, Peter Boknik, Friedrich Köpp, Igor B. Buchwalow, Joachim Neumann, Ulrich Gergs

**Affiliations:** 1 Institut für Pharmakologie und Toxikologie, Medizinische Fakultät, Martin-Luther-Universität Halle-Wittenberg, D-06097 Halle, Germany; mara.doerner@web.de (M.-F.D.); friedrich.koepp@gmail.com (F.K.); joachim.neumann@medizin.uni-halle.de (J.N.); 2 Mibe GmbH Arzneimittel, D-06796 Brehna, Germany; 3 Institut für Pharmakologie und Toxikologie, Medizinische Fakultät, Westfälische Wilhelms-Universität, D-48149 Münster, Germany; boknik@uni-muenster.de; 4 Institute for Hematopathology, Fangdieckstr. 75a, D-22547 Hamburg, Germany; buchwalow@pathologie-hh.de

**Keywords:** transgenic mice, PP2A, PP5, heart failure, fibrosis, inflammation

## Abstract

As part of our ongoing studies on the potential pathophysiological role of serine/threonine phosphatases (PP) in the mammalian heart, we have generated transgenic mice with cardiac muscle cell-specific overexpression of PP2Acα (PP2A) and PP5 (PP5). For further studies we crossbred PP2A and PP5 mice to obtain PP2AxPP5 double transgenic mice (PP2AxPP5, DT) and compared them with littermate wild-type mice (WT) serving as a control. The mortality of DT mice was greatly enhanced vs. other genotypes. Cardiac fibrosis was noted histologically and mRNA levels of collagen 1α, collagen 3α and fibronectin 1 were augmented in DT. DT and PP2A mice exhibited an increase in relative heart weight. The ejection fraction (EF) was reduced in PP2A and DT but while the EF of PP2A was nearly normalized after β-adrenergic stimulation by isoproterenol, it was almost unchanged in DT. Moreover, left atrial preparations from DT were less sensitive to isoproterenol treatment both under normoxic conditions and after hypoxia. In addition, levels of the hypertrophy markers atrial natriuretic peptide and B-type natriuretic peptide as well as the inflammation markers interleukin 6 and nuclear factor kappa B were increased in DT. PP2A enzyme activity was enhanced in PP2A vs. WT but similar to DT. This was accompanied by a reduced phosphorylation state of phospholamban at serine-16. Fittingly, the relaxation times in left atria from DT were prolonged. In summary, cardiac co-overexpression of PP2A and PP5 were detrimental to animal survival and cardiac function, and the mechanism may involve dephosphorylation of important regulatory proteins but also fibrosis and inflammation.

## 1. Introduction

**Figure 1:**
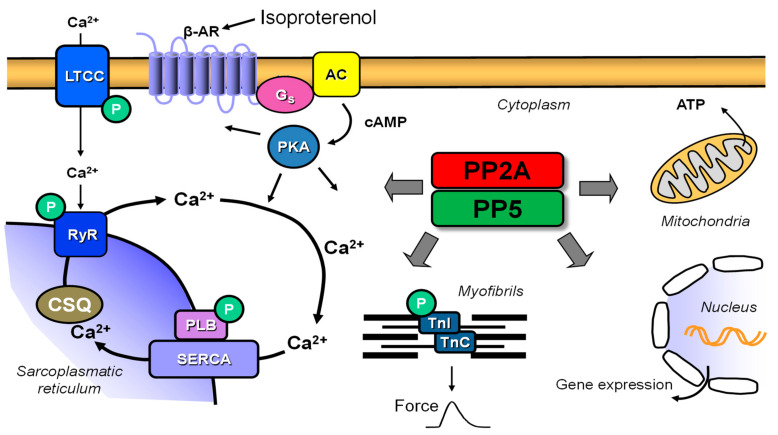
Schematic view of a cardiac muscle cell: isoproterenol acts on the β-adrenoceptor in the sarcolemma where it activates adenylyl cyclases (AC) via stimulatory G-proteins (Gs). This increases cAMP-levels and cAMP activates cAMP dependent protein kinases (PKA). PKA then phosphorylates regulatory proteins such as phospholamban (PLB), the ryanodine receptor (RyR), the L-type Ca channel (LTCC) or troponin I (TnI) in the myofilaments. Finally, Ca^2+^ is elevated, binds to troponin C (TnC) which starts muscle contraction. These phosphorylations are in part reversed by protein phosphatases called PP2A or PP5. See text for further details.

Serine and threonine phosphatases (PP) such as PP1, PP2A, PP2B, PP2C, PP4 and PP5 are present in the cardiomyocytes of mice and humans [1,2,3,4,5]. PP activity is enhanced in some forms of human heart failure and this could be recapitulated in some animal models of hypertrophy and heart failure [6,7]. In addition, PP activity can be related to cardiac arrhythmia [8,9]. Overexpression of PP in mouse hearts, for example, elevated levels of the catalytic subunit of PP1, PP2A, PP2B, PP2C or PP5, have exhibited various degrees of hypertrophy, eventually leading to heart failure [1,10,11,12]. Some of these transgenic mice had higher stress resistance than their wild-type (WT) counterparts in certain experiments [3,13,14,15,16]. Mice with elevated cardiac levels of, for instance, PP1, PP2A or PP5, exhibit typically reduced phosphorylation states of regulatory proteins in various subcellular compartments of cardiomyocytes. The regulatory proteins of interest in this context reside in the sarcolemma, the sarcoplasmic reticulum, the mitochondria and the nucleus of cardiomyocytes (Figure 1) [4]. Reduced phosphorylation states of these regulatory proteins can explain their phenotypes, such as impaired force generation and prolonged relaxation of developed force, to a large extent. Similar observations could also be made in failing human hearts [4,17]. Based on these data, inhibitions of enzymatic activity of phosphatases with cell membrane permeant molecules (tacrolimus for PP2B, okadaic acid for PP1 and PP2A [4,18,19,20]) or proteins that can be delivered by an adenovirus such as inhibitor-2 or inhibitor-1 (for PP1 [1,21,22,23,24,25]) have been suggested to be of a potential benefit in heart failure patients. Whereas about 400 different protein kinases are known to increase the phosphorylation state of regulatory proteins only about 30 protein phosphatases are studied (review in [26]). In evolution, the mechanism of the target specificity and activity of PP becomes clearer. It was found that the best studied protein phosphatase PP1 can regulate their target specificity and activity by more than 40 additional regulatory proteins, including inhibitor-1 and inhibitor-2. Likewise, it is becoming clearer that the activity of the other main cardiac protein phosphatase PP2A is regulated by ancillary proteins [2].

PP2A is member of the PPP family of serine/threonine protein phosphatases [27]. The core enzyme of PP2A is a dimer consisting of a catalytic and scaffolding subunit. In addition, four different forms of another variable regulatory subunit can be distinguished in the core enzyme. These are encoded by different genes and form different splice variants. The three subunits together form the holoenzyme of PP2A. The regulatory subunits range in size from 65 to 108 kDa, are expressed in a tissue-specific manner and determine the localization and substrate specificity of the holoenzyme of PP2A [28]. Its importance in the cardiac function is due to its influence on diverse ion channels, regulatory proteins and transporters and, therefore, it regulates proteins of the contractile apparatus, calcium regulation, cell metabolism, gene regulation, and diverse signaling pathways. This diversity comes from the different structures of PP2A holoenzymes, conditioned by the variable regulatory subunit with its different splice variants, most of which are found in cardiac tissue [29]. The major membrane substrates of PP2A in the heart are the voltage-gated L-type Ca^2+^ channel [30], the Na^+^/Ca^2+^ exchanger, the Na^+^/K^+^-ATPase [31], and connexin 43 [29]. At the sarcoplasmic reticulum, PP2A can dephosphorylate the ryanodine receptor [32,33]. PP2A further decreases the activity of the Ca^2+^ ATPase of the sarcoplasmic reticulum (SERCA) and can dephosphorylate phospholamban (PLB) at serine-16 and threonine-17 [4,34,35,36]. At the myofilaments, PP2A can dephosphorylate the troponin inhibitor complex TnI at serine-23 and serine-24, and possibly also bind to cardiac myosin-binding protein C [37,38,39,40]. In the nucleus, PP2A can dephosphorylate histone deacetylase 4 and controls its subcellular localization, thereby controlling its activity [41,42]. We have previously generated and described transgenic mice with cardiac specific overexpression of PP2A and demonstrated that PP2A serves as a modulator of cardiac function (Figure 1) [10,13].

PP5 is a member of the PPP family of serine/threonine protein phosphatases such as PP1 and PP2A but with the unique feature that catalytic, regulatory and targeting functions are combined on one protein [43]. Additionally, in contrast to other protein phosphatases, PP5 has a N-terminal tetratricopeptide (TPR) domain, which is a regulatory motif for protein–protein interactions [44,45]. PP5 has low phosphatase activity because it is inhibited by the interaction of the TPR domain with an inhibitory region in the catalytic domain itself [46]. This is referred to as autoinhibition within the polypeptide between the N- and C-terminus [27]. PP5 can bind to heat shock protein 90 (HSP90) and is thus involved in glucocorticoid and estrogen receptor function [46,47]. HSP90 can interact with the TPR domain in vivo and resultantly increase phosphatase activity [48,49]. The interaction of the TPR domain with HSP90 and the negative regulation of heat shock factor 1 (HSF1), which is a transcription factor that controls HSP70 and HSP90, among others, is thought to influence PP5 on glucocorticoid receptor-mediated action [27,50]. PP5 likewise affects cell apoptosis processes by being able to inhibit H_2_O_2_-induced activation of ASK-1 (apoptosis signal-regulated serine/threonine kinase-1), thereby preventing the ASK1-dependent signaling pathway of cell apoptosis [14]. Little is known about cardiac functions of PP5. Recently, it was reported that S100 proteins can modulate PP5 functions [51] and S100A1 that amongst others interacts with the cardiac ryanodine receptor and the Ca^2+^ ATPase of the sarcoplasmic reticulum (SERCA), plays an important role in the inflammatory response in cardiomyocytes [52,53]. We have previously generated and described transgenic mice with cardiac muscle cell-specific overexpression of PP5 and demonstrated that PP5 serves as a modulator of cardiac function [11] and inflammation (Figure 1) [3]. Moreover, PP5 in association with HSP90 interacts with the sarcomeric mechanosensor complex and regulates titin phosphorylation and function at the myofilaments of cardiac myocytes [54]. 

In the present work, the effects of co-overexpression of the catalytic subunit of PP2A and PP5 on cardiac performance, taking into account stress parameters such as β-adrenergic stimulation and hypoxia, as well as age, were investigated. Parts of the results have been presented and published as congress abstracts [55,56,57,58,59].

## 2. Results

### 2.1. Phenotype of Transgenic Mice

**Figure 2:**
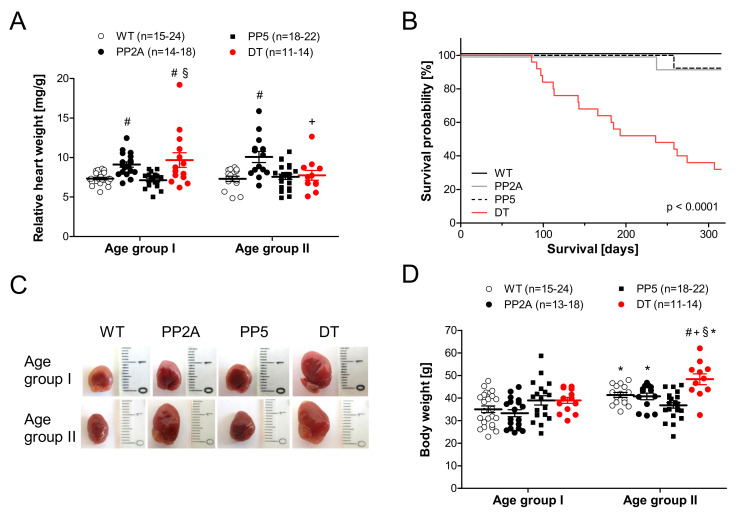
Cardiac gravimetry and survival. (**A**): Relative heart weight (heart weight/body weight quotient) of young (age group I) and middle-age (age group II) mice, (**B**): Time dependent survival of WT, PP2A, PP5 and DT mice. WT: *n* = 10; PP2A: *n* = 13; PP5: *n* = 13; DT: *n* = 25. (**C**): Photography of typical hearts of mice of both age groups from the four genotypes studied, (**D**): body weight of young and middle-age mice, # *p* < 0.05 vs. WT; + *p* < 0.05 vs. PP2A; § *p* < 0.05 vs. PP5; * *p* < 0.05 vs. age group I.

**Figure 3:**
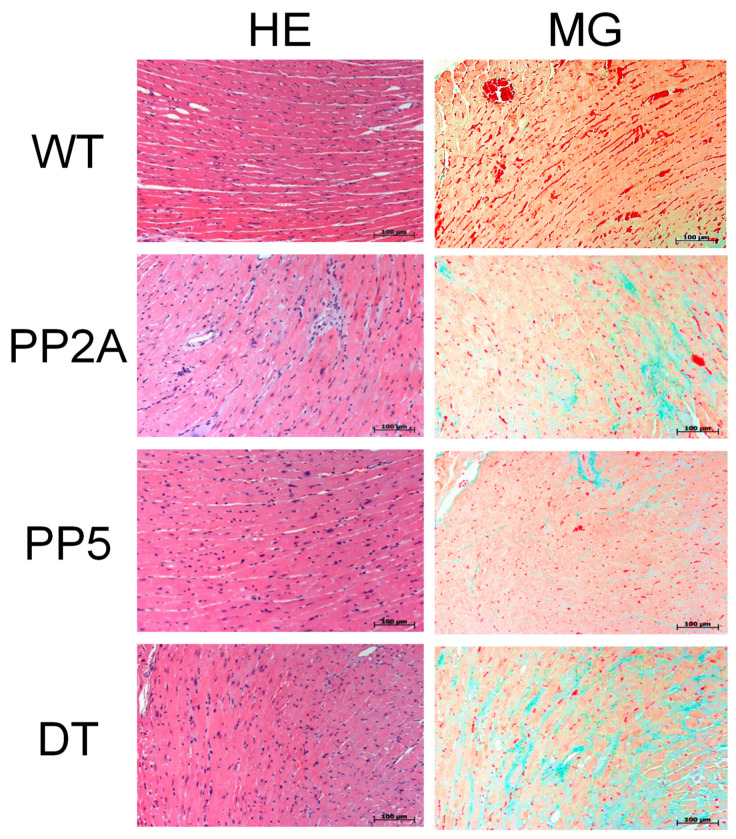
Hematoxylin-Eosin-staining (HE) and Masson-Goldner-trichrome-staining (MG) of heart slices from WT, PP2A, PP5 and DT mice of age group II.

As depicted in Figure 2A, the relative heart weight was higher in PP2A mice than in WT or PP5 mice, and in addition, DT had a larger relative heart weight than WT or PP5 in mice of age group I (Figure 2A left-hand side). However, in older DT mice (age group II), the relative heart weight was smaller than in PP2A mice (Figure 2A right-hand side) because of the increased body weight in DT mice with age (Figure 2D). Typical photographs of the hearts of young and middle-age mice are shown in Figure 2C. They confirm the cardiac hypertrophy of DT mice that was present in age group I mice and still preserved in the older age group II mice. The photographs further illustrate severely hypertrophic atria in DT. In addition, lung weight in younger DT mice was increased compared to the other genotypes (Table 1). The question of how these mice are different in their life expectancy arose here. Interestingly, in DT mice, the survival was worse than in PP2A and PP5 mice. PP2A and PP5 mice did not exhibit reduced survival compared with WT (Figure 2B). From some hearts, exemplary histological slices were prepared to get a general overview of the cardiac morphology and fibrosis. Histologically, no gross alterations between phenotypes were noted in hematoxylin-eosin staining; however, in Masson Goldner trichrome staining, signs of fibrosis were notable in younger DT animals (data not shown), which became more apparent in middle-age animals of DT and of PP2A, too (Figure 3).

### 2.2. Expression of PP2A and PP5

**Figure 4:**
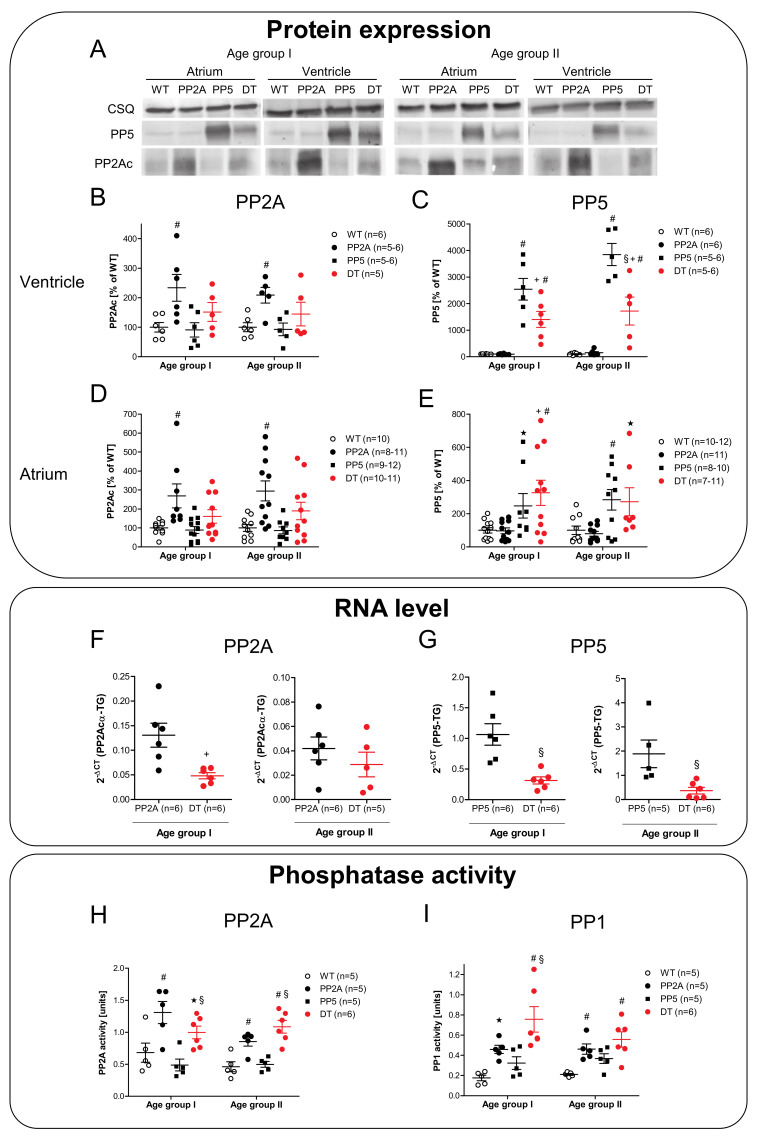
Protein expression of PP2Ac and PP5 in ventricles and left atria of young (age group I) and middle-age (age group II) mice. (**A**): Typical Western Blots, Calsequestrin (CSQ) as loading control; (**B**): PP2Ac-expression in ventricle; (**C**): PP5-expression in ventricle; (**D**): PP2Ac-expression in left atrium; (**E**): PP5-expression in left atrium. mRNA level of PP2Ac and PP5 in ventricles of young and middle-age mice. (**F**): plot of cardiac mRNA density of PP2A in young and middle-age mice, (**G**): plot of cardiac mRNA density of PP5 in young and middle-age mice. Enzymatic activity against phosphorylase a of PP1, PP2A in ventricles of young and middle-age mice. (**H**): Activity of PP2A; (**I**): Activity of PP1. # *p* < 0.05 vs. WT; + *p* < 0.05 vs. PP2A; § *p* < 0.05 vs. PP5; * *p* < 0.05 vs. WT (Students *t*-test); *n* = 4–12.

Next, it was of interest to assess the level of protein overexpression of PP. As seen in the representative original Western blots, the patterns of overexpression were comparable in the atrium and ventricle (Figure 4A). PP2Ac was overexpressed in PP2A mice compared to WT and PP5 (Figure 4B,D), but interestingly in DT, the protein expression of PP2Ac was only slightly increased compared to WT (without significance). However, PP5 was overexpressed in PP5 and DT compared to WT and PP2A in atria and ventricles (Figure 4C,E). Again, to our surprise, the overexpression in DT mice was less than in middle-age PP5 mice (Figure 4C). Moreover, the overexpression did not differ between young and older mice. A somewhat different pattern emerged on the mRNA level: in younger mice, the mRNA for the PP2Acα-TG was higher in PP2A mice compared to DT mice (Figure 4F, left-hand side). In older mice, this difference vanished (Figure 4F, right-hand side). A different pattern was observed for PP5. The mRNA for the PP5-TG was higher in PP5 mice compared to young and middle-age DT mice (Figure 4G). Furthermore, the PP activity in the hearts of the mice was determined. Interestingly, independent of the reduced PP2A protein overexpression observed in DT hearts, the PP2A activity (Figure 4H) as well as the PP1 activity (Figure 4I) were increased to the same amount in young and middle-age PP2A and DT mice compared to WT or PP5 mice.

### 2.3. Atrial Measurements

**Figure 5:**
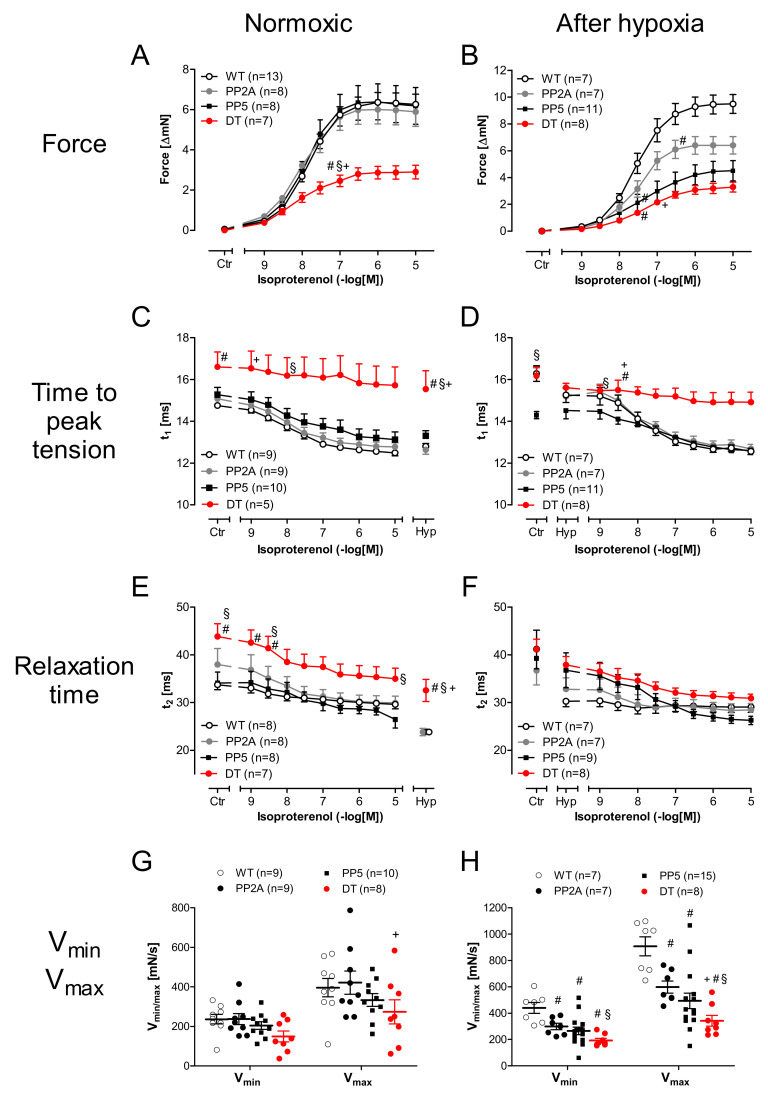
Cumulative concentration response curves of isoproterenol in isolated paced (1 Hz) atrial preparations under normoxic control conditions (**A**,**C**,**E**,**G**) or after hypoxia (**B**,**D**,**F**,**H**)) in young (age group I) mice. (**A**,**B**): Cumulative concentration response curves of isoproterenol on force of contraction; (**C**,**D**): Cumulative concentration response curves of isoproterenol on time to peak tension. (**E**,**F**): Cumulative concentration response curves of isoproterenol on relaxation time. # first significant difference *p* < 0.05 vs. WT; + first significant difference *p* < 0.05 vs. PP2A; § first significant difference *p* < 0.05 vs. PP5; *n* = 5–12. (**G**,**H**): Cumulative concentration response curves of isoproterenol on minimum (V_min_) and maximum (V_max_) rate of tension development. # *p* < 0.05 vs. WT; + *p* < 0.05 vs. PP2A; § *p* < 0.05 vs. PP5; *n* = 8–16.

In isolated left atrial preparations of the younger mice (age group I), we noted an impaired positive inotropic effect of isoproterenol in DT (Figure 5A). Likewise, the time to peak tension was prolonged in DT, and isoproterenol was not able to shorten this parameter to the values measured in the other three genotypes investigated (Figure 5C). Similarly, the time of relaxation was longer in DT compared to WT and PP5. This parameter was shortened by isoproterenol in DT but did not reach the smaller values of the other genotypes (Figure 5E). Under the conditions of isoproterenol followed by hypoxia, the maximum and minimum rate of tension development were determined at the end of the hypoxia. The maximum rate of tension development (V_max_) as well as the minimum rate of tension development (V_min_), a measurement of the ability of a muscle to relax under isometric conditions, was unchanged between genotypes (Figure 5G).

**Figure 6:**
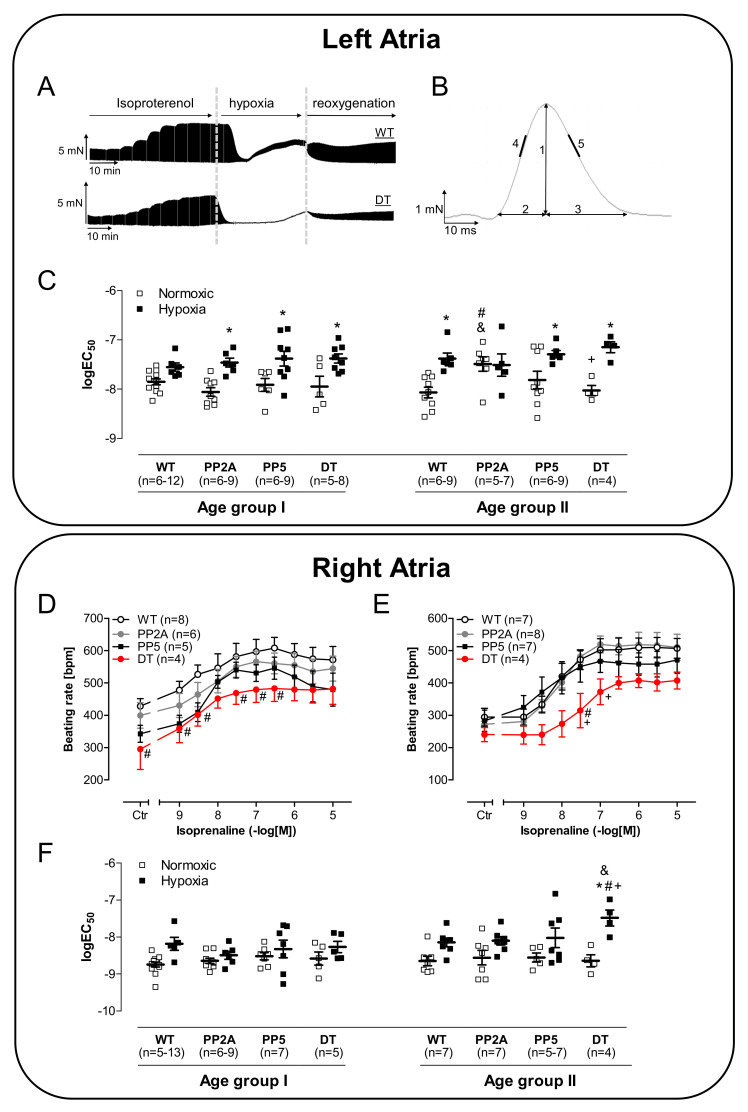
Typical original recordings of the contraction force in left atria (**A**) and the evaluated ranges of the peak (**B**) and corresponding logEC_50_-values on cumulative concentration response curves of isoproterenol of isolated left atrial preparations on contraction force of young (age group I) mice and middle-age (age group II) mice (**C**). # *p* < 0.05 vs. WT; + *p* < 0.05 vs. PP2A; § *p* < 0.05 vs. PP5; & *p* < 0.05 vs. age group I, *n* = 4–12. Cumulative concentration response curves of isoproterenol on beating rate in isolated spontaneously beating right atrial preparations under normoxic control conditions (**D**) or after hypoxia (**E**) in middle-age mice and corresponding logEC_50_-values (**F**). # *p* < 0.05 vs. WT; + *p* < 0.05 vs. PP2A; & *p* < 0.05 vs. age group I; * *p* < 0.05 vs. normoxic; *n* = 4–13.

However, in atrial preparations that undergo first a period of hypoxia followed by isoproterenol, a diminished positive inotropic effect of isoproterenol was noted in PP5 and DT compared to both, WT and PP2A, whereby the positive inotropic effect of PP2A was also decreased compared to WT (Figure 5B). Whereas the peak to tension was prolonged in DT (Figure 5D), no differences in relaxation time were detected (Figure 5F). However, the maximum peak of tension development at the highest concentration of isoproterenol was less in PP2A and PP5 than in WT; DT fared even worse (Figure 5G, left-hand side). Similar, the minimum rate of tension development was found to be increased by isoproterenol but less in PP2A and PP5 compared with WT and even less in DT compared with all other genotypes (Figure 5G, right-hand side). Typical original recordings of the contraction force in left atria and the evaluated ranges of the peak are shown in Figure 6A,B. The EC_50_-values (molar drug concentration for the half-maximum effect) of isoproterenol in left atria demonstrate a decreased positive inotropic effect by hypoxia in all transgenic mice. Interestingly, in the older PP2A, the positive inotropic effect was decreased equally under normoxic and hypoxic conditions (Figure 6C).

Whereas the positive chronotropic effect to isoproterenol in the isolated right atrial preparations of the studied mice under normoxic conditions was similar in all genotypes (data not shown), the beating rate was diminished in young DT (Figure 6D) and in older DT (Figure 6E) after hypoxia. Of note, in middle-age DT, the positive chronotropic effect after hypoxia was altered: the EC_50_-value was significantly lower than in the other genotypes (Figure 6F).

### 2.4. Ventricular Function

**Figure 7:**
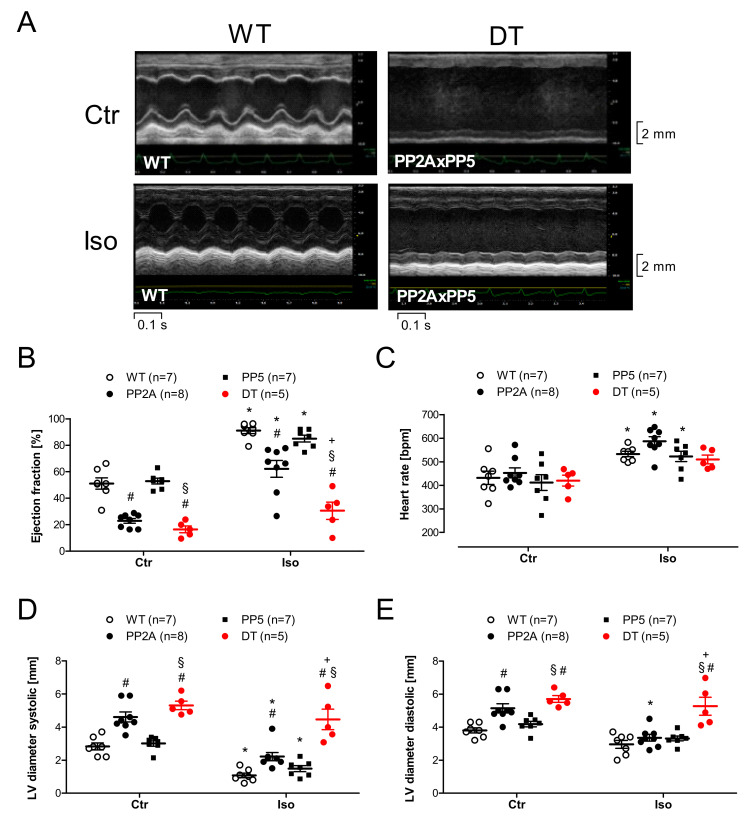
M-mode echocardiography with original recording under basal conditions (Ctr) and after isoproterenol (Iso) in the young mice (age group I). (**A**): Original M-mode recording of WT and DT in young mice; (**B**): Ejection fraction; (**C**): Heart rate; (**D**): Left ventricular (LV) systolic diameter; (**E**): Left ventricular (LV) diastolic diameter; # *p* < 0.05 vs. WT; + *p* < 0.05 vs. PP2A; § *p* < 0.05 vs. PP5; * *p* < 0.05 vs. Ctr; *n* = 5–9.

**Figure 8:**
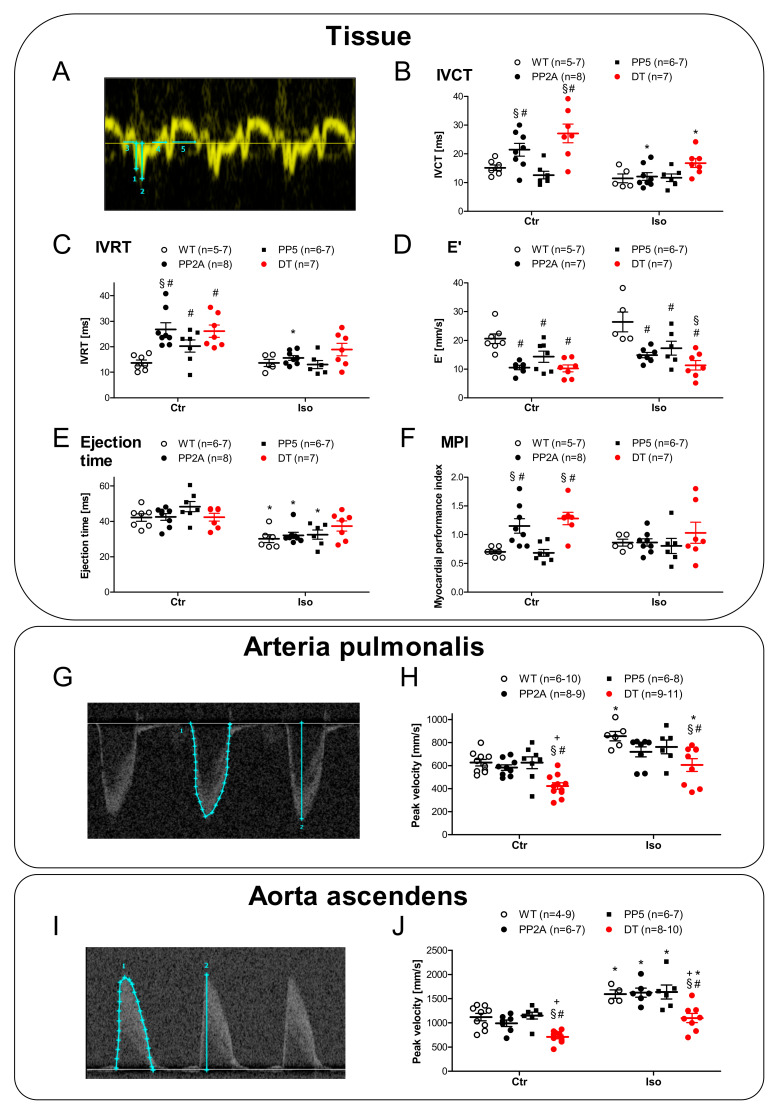
Typical original tracings of Doppler-echocardiography in young (age group I) mice before (Ctr) and after application of isoproterenol (Iso). (**A**): Original recording of tissue Doppler; (**B**): isovolumetric contraction time (IVCT); (**C**): isovolumetric relaxation time (IVRT); (**D**): early diastolic velocity of the mitral anulus (E′); (**E**): ejection time; (**F**): myocardial performance index (MPI); (**G**): Typical original recording of Doppler-mode in arteria pulmonalis; (**H**): Peak velocity in Arteria pulmonalis; (**I**):Typical original recording of Doppler-mode in Aorta ascendens; (**J**): Peak velocity in Aorta ascendens; # *p* < 0.05 vs. WT; § *p* < 0.05 vs. PP5; + *p* < 0.05 vs. PP2A; * *p* < 0.05 vs. Ctr; *n* = 4–11.

Compared to the atrial function, it is obviously relevant how cardiac function is altered in the ventricle and in the intact mouse because these measurements might monitor the clinical situation more faithfully. Therefore, echocardiographic measurements were performed. Original recordings of the parasternal long-axis view in WT and DT mice under basal conditions and after induction of cardiac stress by β-adrenergic stimulation with isoproterenol are shown in Figure 7A. Here, we noted some substantial impairments of left ventricular ejection fraction (EF) in PP2A and DT compared to WT but not in PP5 compared to WT under basal conditions (Figure 7B, left-hand side). Interestingly, the differences between DT and the other genotypes became more visible under substantial burden after β-adrenergic stimulation: the isoproterenol-mediated increase in EF was nearly eliminated in DT compared to all other genotypes (Figure 7B, right-hand side). By this way, it could be demonstrated that β-adrenergic stimulation (sympathetic activation) was not suitable to increase the inotropy of the failing hearts in DT. Moreover, the beating rate was less responsive to isoproterenol in DT in contrast to the other genotypes (Figure 7C). In PP2A and DT mice, left ventricular systolic and diastolic dimensions were found elevated under basal conditions (Figure 7D,E left-hand side). Under β-adrenergic stimulation by isoproterenol, ventricular dimensions appeared normalized in PP2A but remained worse in DT (Figure 7D,E right-hand side). A similar pattern holds true if one looks to the tissue Doppler measurements (Figure 8A) and especially to the early diastolic mitral annular velocity (E′). Here again, even under basal conditions, PP2A and DT were impaired, and after isoproterenol, PP5 exhibited a detrimental increase in E′ compared to WT (Figure 8D). A detrimental function in a more isolated pattern was visible in the ejection time. EF was not shortened after isoproterenol in DT but in all other genotypes (Figure 8E). Similarly, the cardiac isovolumetric contraction time (IVCT) was larger in PP2A and DT than in WT or PP5. IVCT was found to be shortened less in DT after isoproterenol injection (Figure 8B). A somewhat comparable result was noted in the cardiac isovolumetric relaxation time (IVRT): here, PP2A was even more impaired than DT (Figure 8C). A similar impairment of cardiac function was seen in the myocardial performance index (MPI). It was higher in PP2A and DT than in WT or PP5 (Figure 8F). Next, the flow through the aorta ascendens and the arteria pulmonalis was measured. Typical original recordings and the way we quantified these recordings are shown in Figure 8G,I. Under basal conditions, the peak flow in the arteria pulmonalis was smaller in DT than in the other genotypes (Figure 8H, left-hand side) and isoproterenol was able to increase the peak flow in WT and in DT but not in PP2A and PP5 (Figure 8H, right-hand side). A similar but not identical pattern was found in the aorta ascendens: under basal conditions, peak flow was smaller in DT than in all other genotypes (Figure 8J, left-hand side). However, in this case, isoproterenol was able to increase the peak flow through the aorta in all genotypes (Figure 8J). Nevertheless, the peak flow in the aorta ascendens after isoproterenol was smaller in DT than in the other genotypes (Figure 8J, right-hand side). Finally, the echocardiographic measurements can be roughly summarized as follows: PP5 mice mostly behave similar to WT. The cardiac function of PP2A mice was impaired compared to WT (and PP5) but the cardiac function of DT mice was further deteriorated compared to all other genotypes. Interestingly, the cardiac function was not further deteriorated by age as shown exemplarily for the ejection fraction (Table 2).

### 2.5. mRNA Expression

**Figure 9:**
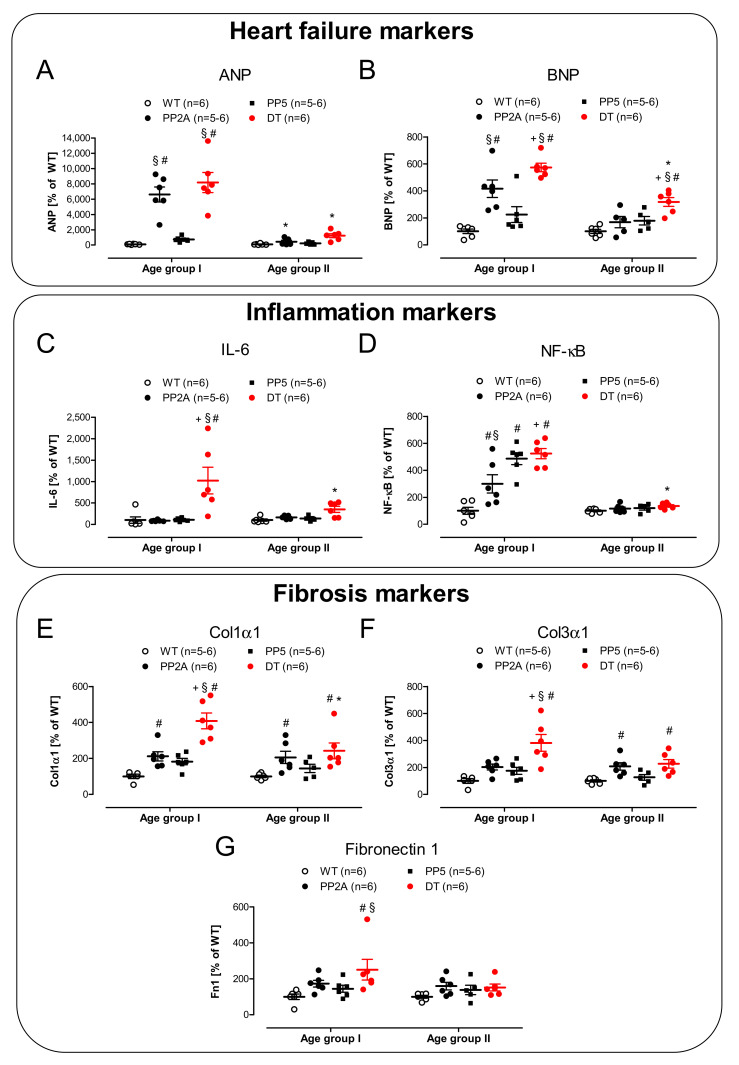
2^−∆CT^-plot of cardiac mRNA density of markers of heart failure, inflammation and fibrosis in young (age group I) and middle-age (age group II) mice. (**A**): atrial natriuretic peptide (ANP); (**B**): B-type natriuretic peptide (BNP); (**C**): interleukine-6 (IL-6); (**D**): Nuclear factor kappa B (NF-κB); (**E**): collagen type I, alpha 1 (Col1α1); (**F**): collagen type 3, alpha 1 (Col3α1); (**G**); fibronectin 1 (Fn1); # *p* < 0.05 vs. WT; + *p* < 0.05 vs. PP2A; § *p* < 0.05 vs. PP5; * *p* < 0.05 vs. young; *n* = 5–6.

As major markers of heart failure, we have chosen to measure the mRNAs of the atrial natriuretic peptide (ANP) and the B-type natriuretic peptide (BNP). The biological functions of natriuretic peptides and their involvement in the regulation of, e.g., blood pressure have been investigated for a long time (review in, e.g., [60,61]). The cardiac expression of ANP and BNP was found to be up-regulated in several kinds of heart disease including cardiac hypertrophy, cardiomyopathies, and mechanical stress (review in [62,63,64]). Consistent with our echocardiographic findings, we noted higher mRNA levels for ANP and BNP in PP2A mice and DT, at least in mice of age group I (Figure 9A,B, left-hand side). However, in older hearts (age group II), ANP and BNP remained elevated only in DT mice (Figure 9A,B, right-hand side). Heart failure has often been shown to be correlated with inflammation [65]. Hence, we attempted to assess inflammation in the heart by measuring mRNA parameters. Here, we noted that some parameters of inflammation were actually elevated. A somewhat selective parameter of inflammation was IL-6; its mRNA was greatly and selectively enhanced in young DT, but this elevation was greatly reduced with time in middle-age DT (Figure 9C). Whereas no significant differences for tumor necrosis factor-α (TNF-α) were noted (data not shown), nuclear factor kappa B (NF-κB) increased in young mice with genetically modified PP (Figure 9D, left-hand side). This elevation subsided with age (Figure 9D, right-hand side). Fibrosis, which we saw in histology (Figure 3), is also probably visible on the mRNA level. Typical parameters of fibrosis were elevated in DT (Figure 9E–G). Here, similar to above, the differences were more pronounced in young hearts than in older hearts. This was also found for collagen 1α1, collagen 3α1 and fibronectin 1 (Figure 9E–G).

### 2.6. Protein Phosphorylation

**Figure 10:**
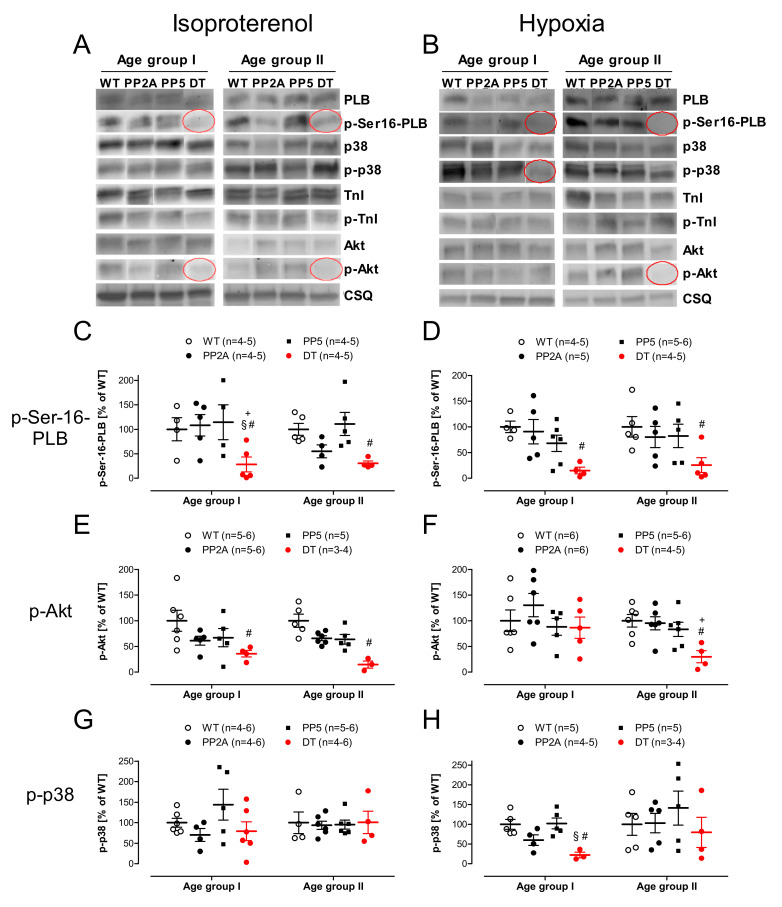
Phosphorylation state of regulatory proteins in atria freeze clamped directly after isoproterenol and directly after hypoxia of young (age group I) and middle-age (age group II) mice. (**A**): Original Western blots after isoproterenol, Calsequestrin (CSQ) as loading control; (**B**): Original Western blots after hypoxia; Calsequestrin (CSQ) as loading control; (**C**): Serine-16 phosphorylation of phospholamban (p-Ser-16-PLB) after isoproterenol, (**D**): Serine-16 phosphorylation of phospholamban after hypoxia; (**E**): Phospho-Akt (p-Akt) after isoproterenol; (**F**): Phospho-Akt after hypoxia; (**G**); Phospho-p38-MAPK (p-p38) after isoproterenol; (**H**): Phospho-p38-MAPK after hypoxia; # *p* < 0.05 vs. WT; + *p* < 0.05 vs. PP2A; § *p* < 0.05 vs. PP5; *n* = 3–6.

The phosphorylation of some proteins was measured in left atria that were treated with isoproterenol and deep-frozen at the highest concentration of isoproterenol. That is accompanied physiologically by the maximum phosphorylation of regulatory proteins. For exemplary Western blots see Figure 10A. In those atria the phosphorylation of Akt and phospholamban at serine-16 in young and middle-age DT mice was reduced compared with the other genotypes (Figure 10C,E). The phosphorylation of p38-MAPK and troponin I (TnI, data not shown) was similar in all genotypes (Figure 10G). In atria, which were frozen directly after hypoxia (for exemplary Western blots see Figure 10B), similarly, the phosphorylation of serine-16 was decreased in young and middle-age DT mice (Figure 10D) and Akt phosphorylation was reduced in the older DT mice (Figure 10F, right-hand side). In contrast, the phosphorylation of p38-MAPK was also reduced in young DT mice.

## 3. Discussion

We have shown before that when PP2Ac is overexpressed, this leads to hypertrophy over time [10]. This effect was also detected, but to a milder extent, in PP5 overexpressing mice [11]. Here, we tested the hypothesis that the effects of one PP can influence the effects of the other PP. Indeed, we noted a greatly deteriorated phenotype in DT mice. More specifically, the heart weight increased substantially in DT. Of note, the body weight in young DT was similar to WT. This could indicate a diseased state in DT compared with PP2A or PP5, which might be a plausible interpretation because the EF was small in DT, and this might lead to increased lung edema, measurable here in increased lung weight in DT. Indeed, relative heart weight has limited value for assessing cardiac hypertrophy when body weight changes with age, but the photographs of DT mouse hearts illustrate the strong cardiac hypertrophy in young and middle-age DT mice. A reduction in EF is clearly functionally relevant because the survival was found to be impaired in DT mice while PP2A and PP5 mice showed no significant impairment in life expectancy in this set of experiments, which is in agreement with our previous publications on these animals [3,10,11]. Histological data indicate fibrosis in the older DT and PP2A mice. Even though the fibrosis was not quantified, which was also not the intended goal of the histology; these data are plausible and might explain in part the problems these hearts have in relaxing. This impairment of relaxation can be clearly seen on isolated tissues and in intact animals. More specifically, impaired relaxation is obvious as a prolonged relaxation time in left atrial preparations under basal conditions, but it becomes more apparent under β-adrenergic conditions, which are well-known in the literature [66]. Of note, the impaired shortening of the time of relaxation and the impaired minimum rate of relaxation after stimulation with isoproterenol all are consistent with impaired cardiac relaxation, even in the left atrium. This is found in a clinical situation only in the late stages of heart failure. Likewise, this is consistent with the impaired Tei-Index (MPI) in DT, an indicator of global impairment of cardiac relaxation [67].

As concern hypoxia as a stressor for cardiac function, there is prior evidence that it is related to PP2A expression. For instance, in experimental hypoxia, the expression of PP2A on protein level increased in the brain of neonatal piglets [68]. In ischemic human hearts, the expression of PP2Ac was found to be enhanced [2]. Likewise, after five days of coronary occlusion in dogs, the expression of PP2Ac was increased in the ischemic cardiac area [2]. At least in AC16 cells (an immortalized cell line of ventricular cardiomyocytes), prolonged hypoxia can lead to decreased expression of PP2Ac and PP2A enzyme activity [69]. In the present work, we addressed this issue by another approach: we genetically increased PP2Ac expression and then asked how this would alter any tolerance for hypoxia. As seen in the EC_50_-values in Figure 6C, in middle-age PP2A mice, the atria could be regarded as protected against a loss of sensitivity to isoproterenol-stimulation, this protection was absent in younger animals and in DT animals as well as PP5 mice. One can, at present, only speculate that PP2A might alter the phosphorylation state of the β-adrenoceptor, which has been reported at least in vitro [70]. This issue needs to be elucidated in subsequent studies. 

The reduced positive inotropic effect of isoproterenol in the left atrium is a classic sign of systolic heart failure in humans, dating back at least to the seminal paper of Michael Bristow [71]; this sign is usually explained by reduced density of β-adrenoceptors in heart failure and a putative protective mechanism for cardiac survival [72]. The longer time to peak tension in DT mice might be explained as follows: less phosphorylation of regulatory proteins under basal and stimulated conditions might indicate less phosphorylation of Ca^2+^ channels and less Ca^2+^ in the cytosol as well as less storage of Ca^2+^ in the sarcoplasmic reticulum during diastole; this means it would take longer to complete a cardiac circle. This is consistent with a reduced increase in the rate of tension development because of isoproterenol. The positive chronotropic effect of isoproterenol was found to be unaltered in young DT mice but altered in the older DT mice, which might indicate that a different compartment of cAMP is present in the sinus node, which is in line with current thinking (review in [73]).

In a clinical situation, we usually assess the function of the left ventricle because it is crucial for the maintenance of the circulation. To assess the systolic function and to diagnose diastolic heart failure, EF is typically assessed. Therefore, we started to measure EF in our animal models resulting in a diminished EF in PP2A and DT mice to similar low levels indicative for a systolic cardiac dysfunction. Moreover, we injected intraperitoneally isoproterenol because this is known to make it easier to detect smaller impairment of cardiac function, which might be masked under basal conditions and can give us a measure of the exercise capacity of the heart under defined stressful conditions. Here, the M-mode echocardiography of the hearts of the three genotypes is very telling. It is apparent that the systolic function of DT hearts is impaired under basal conditions and that they even more badly than PP2A hearts do not adequately respond to sympathetic activation here simulated by injection of isoproterenol (Figure 7B). Incidentally, it seems obvious that this concept of increasing cAMP to sustain cardiac inotropy is not a suitable therapeutic option for this kind of heart failure. This pattern is substantiated by the quantitative measurements of several echocardiographic parameters. Moreover, the data are consistent with our previous work, which indicated that PP5 mice exhibit a milder reduction in systolic and diastolic cardiac performance than PP2A mice [3,10,11]. Here, the difference found in DT mice is striking: they nearly always perform worse (in systolic and diastolic function) than PP5 and even than PP2A mice and clearly always show diminished performance compared with WT. However, the echocardiographic data are also consistent with the initial data in isolated right atrial preparations: in the intact animal much like in the organ bath, the positive chronotropic effect of isoproterenol is blunted.

As a read out or consequence of impaired left ventricular function, we were interested to measure the functional role of the blood flow in arteries. Here, the same pattern as previously found emerged: peak flow in DT mice in the aorta ascendens fare worse than all other genotypes tested. Moreover, blood flow measurements also offered the indirect possibly to measure the function of the right ventricle, which we cannot easily assess in our echocardiographic measurements because it is so small in the mouse and often is hidden behind other structures. However, as a surrogate parameter for right ventricular performance, we measured flow through the arteria pulmonalis, which we found to be reduced in DT. 

As expected, PP2Ac and PP5 were higher in PP2A mice or PP5 mice than in WT mice on the protein level and mRNA level, much like we published for these mice before [3,10,11,74]. A novel finding was a combined overexpression of PP2Ac and PP5 in DT. Interestingly, RNA and protein overexpression of PP2Ac and PP5 are lower in DT than in monotransgenic PP2A or monotransgenic PP5 mice. This effect can be assumed to be specific because in a different double transgenic model, the PP2A mice were crossbred with 5-HT_4_ receptor overexpressing mice and in these double transgenic mice, PP2A expression was unchanged compared to the monotransgenic PP2A mice [75]. Hence, some interesting interaction could exist. It is known that PP5 can bind to A and B subunits of the PP2A holoenzyme, for example to the PR65 and PR72 subunit of PP2A [76]. Moreover, via binding to PP2A B-subunits, PP5 is involved in the regulation of P-glycoprotein expression and function [77] or NF-κB activation [78] or cell cycle progression [79]. This in turn alters the function of PP5. Thus, we speculate that interaction of substrates for PP5 and PP2A occurs and this might alter the stability of the RNA and/or the protein in DT. Other actions and interactions might take place on the gene transcription factors because both PP5 and PP2A dephosphorylate transcription factors, for example p53 [80,81]. Currently, the details of possible interactions in our DT mice remain unclear but should be addressed by further studies.

Cardiac hypertrophy as a compensatory mechanism to keep the heart in a compensated dysfunction state is usually accompanied by altered biochemical parameters, some of which are used clinically to assess the severity of cardiac dysfunction in patients. Consistent with this previous knowledge, it is plausible that the mRNAs for ANP and BNP are elevated in DT hearts. Interestingly, the levels of ANP/BNP mRNAs and others are low in the age group II compared to age group I. One could speculate that in the age group I of PP2A and DT mice, a counter-regulation occurs to compensate the cardiac dysfunction induced by the increased phosphatase activity. The compensatory mechanisms seem to be at least in part successful, which is demonstrated by the transient increase in the tested marker genes. Therefore, the mice develop a compensated cardiac hypertrophy with a final state already reached in the age group I (functional parameters were not further diminished in the group of middle-aged mice) but the transition to decompensated heart failure is prevented. On the other hand, it can at present only be speculated why the mortality of the DT increased. This remains currently unclear; because of technical limitations, e.g., telemetric ECG recordings were unavailable, it was not possible to determine the cause of the death (systolic failure or arrhythmias).

Our data are consistent with the role of inflammation in the initiation of cardiac dysfunction in DT: parameters such as IL-6 are much higher in DT than in the other phenotype, but this elevation goes down with age. Hence, one might speculate that the inflammation drives but does not maintain systolic cardiac dysfunction in DT. To get closer to the mechanism of action of the overexpressed PP in DT, we looked for the known substrates of PP2A and/or PP5 in vitro or in vivo [3,10]. Of special importance is the reduced phosphorylation state and activity of PLB (Figure 10C,D, [35]) under normoxic and hypoxic conditions. Reduced phosphorylation of PLB can easily explain why relaxation is hindered in DT: dephosphorylated PLB inhibits the activity of SERCA [82]; thus, more time is required to pump Ca^2+^ back into the sarcoplasmic reticulum and hence, Ca^2+^ is longer near the filaments; this means that contraction takes longer and relaxation is impaired. The mechanism proposed is consistent with elevated PP2A activity in PP2A and DT mice. One may ask why PP1 activity is increased in PP2A and even more in DT mice. In general, the gene expression profile may be differentially regulated in PP2A and DT mice. However, the increased PP1 activity is consistent with our previous work in PP2A mice, where we noted an increase in PP1 activity because of lower expression levels of I-2, an endogenous inhibitory protein of PP1, as well as reduced phosphorylation, hence creating less action of inhibitor-1 of PP1 (I-1), which we and others have previously reported [7,21,83,84]. In this sense, the increased activity of PP1 may be responsible, at least in part, for the more pronounced phenotype of the DT mice as it is known that cardiac overexpression of PP1 alone is more detrimental compared to PP2A overexpression [1,85]. In summary, we noted that combined overexpression of two phosphatases namely PP2A and PP5 lead to pronounced cardiac hypertrophy and increased mortality, possibly due to the overlapping substrates and regulatory proteins of PP2A and PP5.

Limitations of the study are missing ECG recordings and ventricular hemodynamic measurements. However, it was failed to get stable recordings in DT: hemodynamic measurements are quite stressful for mice and the DT are apparently too frail to allow meaningful measurements. Therefore, these instructive data had to be omitted from the present study. Moreover, using the less invasive method of echocardiography we are able to report on left ventricular function which might overcome this drawback. Furthermore, we measured isometric force of contraction only in atrial preparations and not in ventricular preparations because of the technical limitations. In our experience, the preparations of mouse papillary muscles seldom lead to stable recordings. Here, we would again argue that we have reported on left ventricular functions by performing detailed echocardiography analysis. Another limitation is the missing measurement of the PP5 activity that would allow comparing if the protein und mRNA levels of PP5 correspond to the PP5 activity in DT mice. However, in PP5 mice, the activity assay shows similarly results with PP5 protein expression in PP5 mice [11]. Therefore, we hypothesize that PP5 activity in DT mice might be changed in similar way to the protein levels of PP5. Finally, it is a limitation that it was not possible to answer the question why the ANP/BNP levels get low with age in PP2A and DT mice in spite of a persistent cardiac dysfunction and an increased cardiac hypertrophy. One can even speculate that the decrease on ANP/BNP in middle-aged mice is a biochemical surrogate parameter consistent with a successful compensation of the initial decline in cardiac function in young mice (age group I). Obviously, there are discrepancies between functional (echocardiography) and biochemical diagnoses of heart failure which were beyond the scope of the present study but might be a worthwhile object for subsequent work. Nevertheless, there is a connection of hypertrophy and heart failure in humans that has been reviewed recently (e.g., [86,87]).

## 4. Materials and Methods

### 4.1. Transgenic Mice

All mice used in this study had a genetic CD1 background. Two transgenic lines either with cardiac muscle cell-specific overexpression of rat PP5 [11] or the α-isoform of the catalytic subunit of mouse PP2A (PP2Acα) [10] and as controls their wild-type littermates (WT) were used. Cardiac muscle cell-specific expression was achieved by the use of an expression cassette containing the full length mouse α-myosin heavy chain promoter randomly inserted into the mouse genome by oocyte injection. PP5 transgenic mice were generated utilizing the cDNA of rat PP5 along with 483 base pairs of the 3′ untranslated region as described previously [11]. PP2A transgenic mice were generated utilizing the cDNA of mouse PP2Acα along with 69 base pairs of the 5′ untranslated and 326 base pairs of the 3′ untranslated region as described before [10]. PP2AxPP5 double-transgenic mice were obtained by cross breeding PP2A and PP5 transgenic mice. All transgenic mice were identified by PCR assay of tail genomic DNA. For the experiments, heterozygous mice of each gender were used that were either three to four months old (=young experimental group labeled as “age group I”) or ten to eleven months old (=middle-age experimental group labeled as “age group II”). The results from both genders have been pooled due to the missing differences between male and female mice. The relative heart weight was calculated from the quotient of heart weight/body weight. The animals were handled and maintained according to the approved protocols of the animal welfare committee of the University of Halle-Wittenberg, Halle (approval reference number 42502-02-1518 MLU).

### 4.2. Contractile Studies in Mice

In brief, right or left atrial preparations were isolated and mounted in organ baths as described before [88,89,90]. The bathing solution of the organ baths contained (in mM) NaCl, 119.8; KCI, 5.4; CaCl_2_ 1.8; MgCl_2_, 1.05; NaH_2_PO_4_, 0.42; NaHCO_3_, 22.6; Na_2_EDTA, 0.05; ascorbic acid, 0.28; and glucose, 5.05, continuously gassed with 95% O_2_ and 5% CO_2_ and maintained at 37 °C and pH  7.4 as described. Preparations were attached to a bipolar stimulating electrode and suspended individually in 10 mL glass tissue chambers for recording isometric contractions. Force of contraction was measured with inductive force transducers connected to a chart recorder. Time parameters of single contractions were evaluated at high chart speed. The atrial preparations were electrically stimulated at 1 Hz with rectangular pulses of 5 ms duration; the voltage was ~10–20% greater than threshold. Right atrial preparations were attached in the same set-up but were not electrically stimulated and allowed to contract spontaneously. Contractions were measured in an isometric set-up. Atria were attached with fine sutures to a hook in the organ bath and a isometric force transducer. Signals were amplified and continuously fed into a chart recorder (PowerLab system, ADInstruments, Oxford, UK). For hypoxia measurements, after equilibration time atrial preparations were gassed with 95% N_2_ and 5% CO_2_ for 30 min, followed by 30 min of reoxygenation under normoxic conditions. In the experiments we differ between atrial preparations which undergo an equilibration time (a) followed by a cumulative concentration response curve with isoproterenol from 1 nM to 10 µM and (b) followed by hypoxia, reoxygenation with a subsequent cumulative concentration response curve with isoproterenol from 1 nM to 10 µM. In left atria the contractile parameters were evaluated. The force of contraction was the difference between the maximum and minimum tension at constant muscle length. The change of the force of contraction to the previous status in ΔmN was analyzed. The time from 10% of generated force to peak force was regarded as time to peak tension and the time from peak force to 90% relaxation was termed relaxation time. In right atria the beating rate in bpm was measured.

### 4.3. Echocardiography

Transthoracic echocardiographic measurements in spontaneously breathing mice were performed under anesthesia with 1.5% isoflurane using a Vevo 2100 system equipped with a MS 550D transducer (Visual Sonics, Toronto, ON, Canada). Two-dimensional images and M-mode tracings were recorded from the parasternal long axis view. The cardiac dimensions were measured, and the ejection fraction of the hearts was calculated. In addition, the Doppler option of the Vevo 2100 system was used for arterial flow measurements and tissue measurements, as described previously [3,74]. Ejection fraction of the heart was calculated from the M-mode left ventricular (LV) volume as follows: 100 * (LVEDV-LVESV)/LVEDV, where LVEDV is the LV end-diastolic volume and LVESV is the LV end-systolic volume. The myocardial performance index of the heart was calculated from the tissue Doppler as follows: (IVCT + IVRT)/ET, where IVCT is the iso-volumetric contraction time, IVRT the iso-volumetric relaxation time and ET the ejection time. For experiments under β-adrenergic stimulation, isoproterenol (0.7 mg/kg) was injected intraperitoneal. The use of 1.5% isoflurane as an anesthetic in mice and the intraperitoneal injection of isoproterenol in echocardiogaphy are well established methods and have been routinely used in previous studies [91]. 

### 4.4. Preparation of Homogenates

Frozen ventricular tissue samples were pulverized in a laboratory vibrating mill (Mikro-Dismembrator, Sartorius, Göttingen, Germany) using PTFE vessels and tungsten carbide grinding balls precooled in liquid nitrogen. The following steps were carried out at 4 °C. Then, 300 µL of buffer containing 10 mM NaHCO_3_ and 5% SDS (to prevent proteolysis and dephosphorylation of proteins) were added to the frozen, pulverized tissue. The tissue was then homogenized three times for 30 s each with a Sonopuls ultrasonic homogenizer (Bandelin, Berlin, Germany). Crude extracts were incubated at 25 °C for 30 min before centrifugation (10 min, 14,000× *g*) to remove debris and thereafter, the supernatants (homogenates) were separated and stored at −80 °C until further use. Protein was measured using the Lowry protein assay [92].

### 4.5. Western Blot Analysis

For the Western blot analysis, ventricular homogenates were prepared, and aliquots of 60 μg protein were loaded per lane, as described previously [10]. Protein loading was monitored by Ponceau staining of the nitrocellulose membranes and expression of calsequestrin (CSQ). CSQ was used for normalization of protein expression because it is a marker of cardiac myocytes. Bands were detected using enhanced chemifluorescence (ECF, GE Healthcare, Munich, Germany) and a Typhoon™ 9410 Variable Mode Imager (GE Healthcare, Munich, Germany). The signals were quantified with the ImageQuant TL software (GE Healthcare, Freiburg, Germany). The list of primary antibodies used is summarized in the section titled ‘Drugs and Materials’. Corresponding secondary antibodies conjugated with alkaline phosphatase were purchased from Sigma-Aldrich (Munich, Germany).

### 4.6. Protein Phosphatase Assay 

Phosphorylase phosphatase activity (for PP1 and PP2A) with [^32^P]-phosphorylase as the substrate was determined, as described previously [18]. Portions (20 mg) of pulverized frozen heart ventricular tissue were homogenized at 4 °C three times for 30 s each with a Polytron PT-10 (Kinematica, Luzern, Switzerland) in a 300 µL buffer containing (in mmol/L) TRIS HCl (pH 7.0) 50.0, EDTA 0.1, β-mercaptoethanol 0.1% (*v*/*v*), PMSF 1, benzamidine 1. The homogenate was centrifuged for 20 min at 14,000× *g*. The incubation mixture contained (mmol/L) TRIS HCl (pH 7.0) 20.0, caffeine 5.0, EDTA 0.1 and β-mercaptoethanol 0.1% (*v*/*v*). The reaction was started by adding aliquots of homogenates (containing 3–11 µg protein) or aliquots of peak fractions. The samples were assayed in the presence and absence of 3 nM okadaic acid, which completely inhibits PP2A activity; thus, the remaining activity would only occur because of PP1 (for description see [93]). The reaction was stopped by the addition of 50% trichloroacetic acid. The precipitated protein was sedimented by centrifugation, and the radioactivity in the supernatant was counted in a liquid scintillation counter.

### 4.7. Histological Analysis 

The preparation of cardiac apex samples, mounting and staining was carried out as published before [10]. To analyze the morphological features of transgenic myocardium, dewaxed and rehydrated tissue sections of formaldehyde-fixed ventricular probes were stained with hematoxylin and eosin (HE) and Masson-Goldner trichrome (MG). For imaging a Zeiss Axio Imager Z1 microscope with an AxioCam Digital microscope camera was used and analysed by AxioVision software (Carl Zeiss Vision GmbH, Aalen, Germany). 

### 4.8. Real-Time Quantitative PCR

PCR was carried out as reported before [3]. In brief, the total RNA from pulverized frozen heart ventricular tissue was isolated using the TRIzol^®^ reagent (Invitrogen, Fisher Scientific, Schwerte, Germany) according to the manufacturer’s instructions. Subsequently, reverse transcription was performed using the Maxima First Strand cDNA Synthesis Kit for RT-qPCR with dsDNAse (Fisher Scientific, Schwerte, Germany) according to the manufacturer’s instructions. During cDNA synthesis, the remaining DNA was digested with DNase I. Reverse transcription was performed with 5 μg RNA and a mixture of oligo(dT)18 and random hexamer primers. As a control, each RNA sample was also analyzed without reverse transcription (NRT). Finally, cDNA samples were diluted to a volume corresponding to 10 ng RNA per μL. Real-time PCR amplification and detection was performed with the Bio-Rad CFX Connect Real-Time PCR Detection System using the iTaq Universal SYBR^®^ Green Supermix Kit (BioRad Laboratories, Munich, Germany) according to the manufacturer’s instructions. The relative expression of the genes of interest was calculated according to the 2^−ΔΔCT^ method [94] by using the GAPDH signal for normalisation. The primers either were developed with the software Discovery Studio Gene v1.5 (Accelrys, Cambridge, UK) or were derived from the literature [95,96,97,98]. Primer sequences are summarized in the section titled ‘Drugs and Materials’

### 4.9. Data Analysis

The data shown are means ± SEM. Statistical significance was estimated by an analysis of variance (ANOVA) followed by Bonferroni’s *t*-test or by using the student’s *t*-test when appropriate. A *p*-value < 0.05 was considered significant. Experimental data for agonist-induced positive inotropic and chronotropic effects were analyzed by fitting the sigmoidal curves to the experimental data using GraphPad Prism 5.0. All other statistical analyses were performed as indicated in the figures and tables. The statistical evaluation was conducted with GraphPad Prism 5.0 (GraphPad Software, San Diego, CA, USA), which was also used to produce the graphs. The *n*-numbers in the graph description or directly in the graphs columns give the number of individual hearts studied.

### 4.10. Drugs and Materials 

(−)-Isoproterenol (+)-bitartrate was purchased from Sigma-Aldrich (Deisenhofen, Germany). All other chemicals were of the highest purity grade commercially available. Deionized water was used throughout the experiments. Stock solutions were freshly prepared daily.

Primary antibodies: polyclonal rabbit anti calsequestrin (#ab32141, abcam, Berlin, Germany), monoclonal mouse anti SERCA2a (#ab2861, abcam, Berlin, Germany), monoclonal rabbit anti PP2A alpha and beta (#ab32141, abcam, Berlin, Germany), monoclonal mouse anti PP5 (#611921, BD Biosciences, Heidelberg, Germany), polyclonal rabbit anti Troponin I (#4002, Cell Signaling, Frankfurt am Main, Germany), polyclonal rabbit anti phospho-Ser23/24 TnI (#4004, Cell Signaling, Frankfurt am Main, Germany), monoclonal mouse anti Akt (#2902, Cell Signaling, Frankfurt am Main, Germany), monoclonal rabbit anti phospho-Thr308 Akt (#4056, Cell Signaling, Frankfurt am Main, Germany), polyclonal rabbit anti p38 MAPK (#9212, Cell Signaling, Frankfurt am Main, Germany), monoclonal rabbit anti phosphor-Thr180/182 p38 MAPK (#4511, Cell Signaling, Frankfurt am Main, Germany), monoclonal mouse anti PLB (#A010-14, Badrilla, Leeds, UK), polyclonal rabbit anti phosphor-Ser16 PLB (#A010-12, Badrilla, Leeds, UK).

Primer sequences: ANP, forward, GTGCGGTGCCAACACAGAT, reverse, GCTTCCTCAGTCTGCTCACTCA; BNP, forward, CCAGTCTCCAGAGCAATTCAA, reverse, AGCTGTCTCTGGGCCATTTC; Col1a1, forward, ACATGTTCAGCTTTGTGGACC, reverse, TAGGCCATTGTGTGTATGCAGC; Col3a1, forward, TGGTAGAAAGGACACAGAGGC, reverse, TCCAACTTCACCCTTAGCACC; Fn1, forward, TTAAGCTCACATGCCAGTGC, reverse, TCGTCATAGCACGTTGCTTC; GAPDH, forward, ATGCATCCTGCACCACCAAC, reverse, ATGCCTGCTTCACCACCTTC; IL-6, forward, CCGGAGAGGAGACTTCACAG, reverse, TTCTGCAAGTGCATCATCGT; NFκB, forward, GAAATTCCTGATCCAGACAAAAAC, reverse, ATCACTTCAATGGCCTCTGTGTAG; TNFα, forward, CACACTCAGATCATCTTCTCAAAA, reverse, GTAGACAAGGTACAACCCATCG; Fn1, forward, TTAAGCTCACATGCCAGTGC, reverse, TCGTCATAGCACGTTGCTTC; PP2Acα-TG, forward, ACCCTTACCCCACATAGACC, reverse, CTTAAACACTCGTCGTAGAACC; PP5-TG, forward, ACCCTTACCCCACATAGACC, reverse, GCTTCACCTTCACCACCGTC.

## Figures and Tables

**Table 1:** Organ weights (m = weight) of young (age group I) and middle-age (age group II) mice.

	Age Group I				Age Group II			
	WT	PP2A	PP5	DT	WT	PP2A	PP5	DT
*n*	24	18	20	13	21	19	22	8
m_Lung_ [mg]	220 ± 9	214 ± 8	213 ± 5	262 ± 17 ^#,+,§^	290 ± 7 *	311 ± 10 *	245 ± 8 ^#,^*	273 ± 22
*n*	24	18	20	13	21	19	22	8
m_Liver_ [mg]	1878 ± 83	1694 ± 99	1752 ± 85	2025 ± 99	2310 ± 88 *	2468 ± 162 *	1846 ± 82 ^#^	2695 ± 227 ^§,^*
*n*	11	12	19	13	8	8	22	13
m_Kidney_ [mg]	566 ± 47	523 ± 38	511 ± 39	501 ± 29	587 ± 53	527 ± 48	518 ± 26	573 ± 44

^#^*p* < 0.05 vs. WT; ^+^ *p* < 0.05 vs. PP2A; ^§^ *p* < 0.05 vs. PP5; * *p* < 0.05 vs. age group I.

**Table 2:** Ejection fraction (EF) estimated by echocardiographic under basal conditions and after β-adrenergic stimulation by isoproterenol (Iso) in young 3–4 months old mice (age group I) compared to the middle-age 10–11 months old mice (age group II).

	WT (*n* = 7)		PP2A (*n* = 8)		PP5 (*n* = 7)		DT (*n* = 5)	
EF [%]	Age Group I	Age Group II	Age Group I	Age Group II	Age Group I	Age Group II	Age Group I	Age Group II
**Basal**	51.1 ± 4.31	51.5 ± 3.35	22.6 ± 1.60 ^#^	24.9 ± 2.31 ^#^	51.5 ± 2.4	48.6 ± 3.99	19.0 ± 3.2 ^#,§^	19.8 ± 3.2 ^#,§^
**Iso**	91.1 ± 2.26 *	92.4 ± 1.28 *	62.1 ± 6.33 ^#,^*	61.0 ± 4.21 ^#,^*	85.0 ± 2.56 *	79.0 ± 4.53 *	30.5 ± 6.55 ^#,+,§^	32.2 ± 5.10 ^#,+,§^

^#^*p* < 0.05 vs. WT; ^+^ *p* < 0.05 vs. PP2A; ^§^ *p* < 0.05 vs. PP5; * *p* < 0.05 vs. basal.

## Data Availability

The data presented in this study are available on request from the corresponding author.
